# Prevalence of Bovine Hydatidosis in Cattle Slaughtered at Nekemte Municipal Abattoir, Western Ethiopia

**DOI:** 10.1155/2024/4978078

**Published:** 2024-10-30

**Authors:** Abdu Muhammed, Yobsan Tamiru, Felmata Kenei, Nezif Zenu

**Affiliations:** ^1^Department of Animal Science, Faculty of Agriculture, Wollega University, Shambu Campus, P.O. Box 38, Shambu, Ethiopia; ^2^School of Veterinary Medicine, Wollega University, P.O. Box 395, Nekemte, Ethiopia

**Keywords:** abattoir, cattle, hydatidosis, Nekemte, organ, prevalence

## Abstract

Hydatidosis (cystic echinococcosis) is one of the major serious parasite infectious diseases that cause poor weight gain and organ condemnation, which contributes to Ethiopia's low cattle industry production. A cross-sectional study was performed at the Nekemte Municipal Abattoir, Western Ethiopia, from October 2020 to August 2021 with the aim of determining the prevalence of bovine hydatid cysts, assessing the related risk factors, and evaluating the organ level of distributions of the cysts. An antemortem examination and postmortem examination were performed as usual on all 220 chosen slaughtered cattle. We evaluated the organs of systemically selected cattle through visual inspection and palpation. Of the total number inspected, 44 (20%) had one or more hydatid cysts in one or more of their organs. Both age and sex of the cattle were significantly associated with the prevalence of bovine hydatidosis (*X*^2^ = 5.928; *p* = 0.015; and *X*^2^ = 4.086; *p* = 0.043, respectively) among the risk factors evaluated. 44 (20%) of the 220 animals evaluated were positive for hydatidosis. In terms of organ distribution, the liver accounted for 27 (61.4%), the lung for 16 (36.3%), and the spleen for 1 (2.3%). 34 (55.8%) of the 62 cysts counted and characterized were found in the liver, 27 (53.6%) in the lung, and 1 (2.3%) in the spleen. 22 (35.5%) of the 62 cysts collected were calcified, and 31 (50%) were found to be fertile. Of the 31 fertile cysts discovered, 7 (22.6%) were found in the liver, 23 (74.2%) in the lungs, and 1 (2.3%) in the spleen. Hydatidosis is still one of the most critical diseases that need careful consideration for prevention and control measures in the East Welega Zone, even with the moderate level of infection currently detected. This is because there appears to be a socioeconomic environment that is conducive to the disease. Therefore, the installation of regulated, well-equipped abattoirs, public awareness campaigns, and stray dog control are crucial.

## 1. Introduction

Hydatidosis is one of the significant parasitic diseases causing reduced productivity in meat production due to carcass or organ condemnation. Among the various common livestock diseases, parasitic infections generally pose a severe challenge to livestock development in the tropics generally [1]. Echinococcosis is a zoonotic infection caused by the small tapeworm *Echinococcus granulosus*. In the natural cycle, ungulates such as cattle, sheep, goats, pigs, and horses are intermediate hosts where hydatid cysts develop. Dogs and other canids are typically definitive hosts that excrete parasitic eggs in their feces. These eggs then lead to the development of hydatid cysts in the lungs, liver, and other organs as a result of infection [2, 3]. It is regarded as one of the most common zoonoses in the world, impacting both veterinary and public health [4].

Diagnosing intestinal *E. granulosus* infection in living dogs is challenging because small proglottids spontaneously expelled with feces are often overlooked and because eggs found by standard coproscopics for accuracy cannot be differentiated from eggs of other *Echinococcus* species by light microscopy, and ELISA is used to detect parasite antigens (coproantigens) in fecal samples [5, 6]. Although detecting infection in living animals is difficult, cysts have occasionally been found using ultrasonography alone or in conjunction with serum antibody detection [7].

According to [8], in order to guarantee the safety and wholesomeness of meat for human consumption, postmortem inspection is crucial for meat inspection programs in developing countries. In order to ensure that animal flesh is free from disease and does not present a risk to human health, it is the primary responsibility of public health authorities, veterinarians, and meat inspectors to conduct comprehensive postmortem inspections. For meat inspection in developing countries, antemortem diagnosis of hydatidosis is not useful; instead, postmortem carcass inspection is necessary due to the lack of reliable diagnostic methods, which has led to the widespread spread of the disease [9]. Several options for controlling CE have been thoroughly evaluated and summarized into two basic approaches. One approach is based on legislation aimed at interrupting parasite transmission and long-term public health education with primary health care [10], while the other focuses on veterinary public health activities, such as dog registration, sanitation measures, and improved slaughter hygiene and meat inspection [11].

Hydatidosis is a disease that affects people worldwide, causing significant economic losses and public health issues. It reduces animal output and leads to the rejection of hydatid cysts in slaughterhouses [9]. Despite significant efforts to understand and prevent “cystic echinococcosis, it continues”; it continues to be a global disease with localized outbreaks in specific countries [1]. Infection with *E. granulosus is* found in Central and South America, Eastern and Central Europe, the Middle East, Russia, China, and South and East Africa, including Ethiopia [12].

Several researchers in Ethiopia have observed high hydatidosis prevalence in various regions of the nation [1]. The prevalence of hydatidosis in cattle and small ruminants has been reported to range between 13.1% and 72.44%, respectively [13]. Camels have a prevalence rate of up to 30.8%, whereas dogs have a prevalence rate of up to 25% [14]. Cystic echinococcosis in agricultural animals results in significant losses in meat and milk output, as well as significant economic losses due to organ condemnation and lower meat, milk, and wool quality [9]. The country has claimed economic losses ranging from $3201 to $116,751 USD [14]. These losses are particularly severe in low-income nations where animal production is more important [15].

To prevent or control the diseases, one must be aware of their public health significance, prevalence, and the risk factors linked to them. This information is found in the epidemiology of the disease [12]. The disease is most prevalent in developing countries, especially in rural areas where dogs, the definitive hosts, and other domestic animals, the intermediate hosts, interact frequently. Any attempt to prevent or control the disease requires knowledge of the distribution of the disease to and related risk factors, which make up the epidemiology of the disease [16]. Furthermore, the assessment of the disease's economic impact plays a crucial role in the formulation, organization, and execution of regional management strategies. The prevalence in different regions or countries is the main focus of current research, with less emphasis on the prevalence and particular risk factors in Western Ethiopia. Lack of information impedes the development of focused control tactics, particularly in light of the important roles that animal husbandry techniques, local environmental conditions, and interactions with definitive hosts such as dogs play in the spread of disease. Effective detection and prevention of the disease are further limited by difficulties with diagnosis in live animals and irregularities in postmortem examinations. In order to prevent organ condemnation, reduce economic losses from the disease, and protect public health in the area, further investigation could focus on genotyping of *Echinococcus* strains affecting cattle in different regions of Ethiopia to better understand strain-specific transmission and pathology. Therefore, the objective of this study was to estimate the prevalence, determine potential risk factors, and characterize hydatid cysts in bovine slaughtered at Nekemte Municipal Abattoir, Western Ethiopia.

## 2. Materials and Methods

### 2.1. Study Area

**Figure 1:**
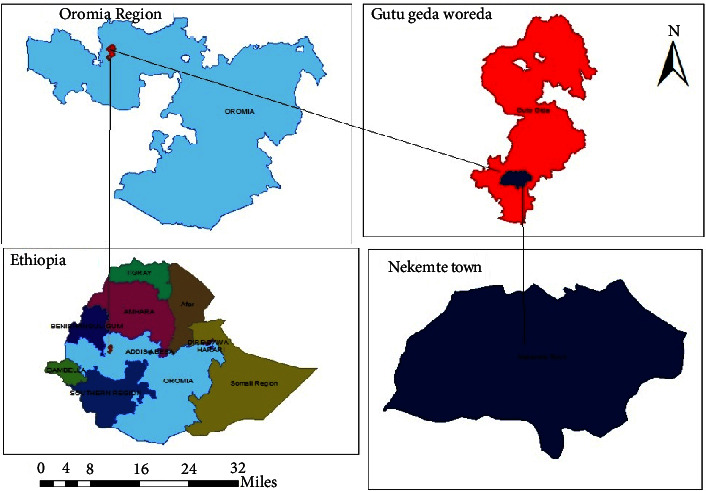
Map showing study areas.

The study was carried out in Nekemte town (Figure 1), located 328 km from Addis Ababa in the East Welega Zone of Oromia Regional State in Western Ethiopia. Nekemte town is situated at an elevation of 2088 m above sea level and lies between latitude 9°05′N and longitude 36°33′E. The region receives 1850 mm of rainfall on average. The average monthly minimum and maximum temperatures were 10.5°C and 31°C, respectively. The livestock population of this zone is approximately composed of 9,25,144 cattle, 1,46,775 goats, 2,20,875 sheep, 92,250 equines, 7,94,484 chickens, and 1,76,532 bee hives. Nekemte Municipal Abattoir is located on the Nekemte-Gimbi main road at Bake Jama kebele (06 kebele). In this abattoir, an average of 35 bovines is slaughtered every day [17].

### 2.2. Study Design and Population

A cross-sectional study was conducted between October 2020 and August 2021 to estimate the prevalence of hydatidosis and its associated risk factors, as well as the distribution of cysts in organs and size of cysts in the organs of cattle slaughtered at Nekemte Municipal Abattoir. The study randomly selected both local and exotic breeds of cattle brought from different areas of the districts, namely, Arjo, Diga, Bandra, Arjo Gudatu, Nekemte town, Getema, Uke, Chewaka, Guder, Wayutuka, Gichian, and Dune Kane. Both male and female animals were slaughtered.

### 2.3. Sampling Method and Sampling Size Determination

The investigation was carried out at Nekemte Municipal Abattoir utilizing a simple random sampling method prior to sample collection, ant-mortem, and postmortem examination for every edible organ of bovine from different locations that were brought in for slaughter. The formula proposed by Thrusfield [18] was used to determine the total number of animals needed for the investigation. To calculate the sample size with a 95% confidence interval (CI) and 5% required absolute precision, the study uses 17.34% predicted prevalence [19].

(1) $$\begin{array}{c} n=\frac{1.96^{2}p\left(1-p\right)}{d^{2}}, \end{array}$$

where *n* = sample size required for study population, *d* = desired precision, and *p* = 50% expected prevalence.

Consequently, the minimum sample size was 220 cattle. They were chosen at randomly from all bovine brought to the Nekemte Municipal Abattoir for slaughter and included in the study.

### 2.4. Study Methodology

#### 2.4.1. Antemortem Examination

According to [20], an antemortem inspection should be performed. The age, sex, and body condition of each animal were recorded. Animals were classified as lean, medium, or good based on their body condition [21]. The age of the animal was assessed based on dentition and was typically categorized into three groups: young (4–6), adult (7–9), and old (> 10 years) [22].

#### 2.4.2. Postmortem Examination

The organs of systematically selected cattle were evaluated by visual inspection and palpation using a postmortem methodology approved by the Food and Agriculture Organization [23]. Following the standard routine post mortem inspection technique, organs of each slaughtered animal affected with hydatid cysts were systematically detected. Additionally, each organ had one or two knife incisions made. If there were cysts, they were extracted, labeled, and sent to the lab for further analysis. They were then stored in separate polyethylene bags [1].

#### 2.4.3. Specimen Collection and Transportation

Each organ of slaughtered animals was examined in the abattoir, particularly the lungs, liver, heart, spleen, and kidneys, using visual inspection, palpation, and, if necessary, one or more incisions [24]. Individual cysts were visually examined for signs of degeneration or calcification, collected, and transported to Wollega University School of Veterinary Medicine, Parasitology Laboratory, where they were kept in an icebox with ice packs for further fertility, sterility, viability, tests, and cyst size determination. The size of the hydatid cyst in the affected organ was determined according to the method proposed in [25–27]. As a result, the cysts were measured systematically and classified as tiny (≤ 4 cm), medium (4–8), or large (≥ 8 cm) in diameter. To minimize pressure, the fluid was aspirated into a sterile cylindrical container with an 18 G needle and a 20 mL syringe. The cyst was incised with a scalpel blade following aspiration, and the entire contents were placed in a cylinder. The germinal layer of the cyst was thoroughly probed for the presence of hydatid sand. The cyst contents were placed in a Petri dish, and a small drop of fluid was transferred to a glass slide, covered with a coverslip, and examined under a low magnification power (40x) to determine the cyst's state [28].

#### 2.4.4. Fertility and Viability Test

The individual cysts were grossly inspected for evidence of degeneration and calcification. Cysts were chosen for fertility studies, and the cyst wall was punctured with a needle and opened with a scalpel blade and scissors to minimize intracystic pressure [29]. The contents were transferred to a sterile container and examined under a microscope (40x magnification) for the presence of protoscolices. Cysts that did not contain any protoscoleces and were excessively suppurative or calcified were considered infertile. The motility of the flame cells as well as staining with 0.1% aqueous eosin solution was used to determine the vitality of protoscoleces. Unlike the dead, living protoscolices did not pick up the stain [30].

#### 2.4.5. Data Management and Analysis

The collected data were analyzed using SPSS version 20. Descriptive statistics, such as percentages and frequency distributions, were used to describe the nature and quality of the data. Associated risk factors were assessed using the chi-square test (*X*^2^). In all analyses, the confidence level was set at 95%, and the significance level was set at 0.05.

## 3. Results

### 3.1. Active Abattoir Survey

During antemortem induction, the associated risk factors (origins, age, sex, breed, and body condition) were documented, and all animals that were inspected were found to be normal and fit for slaughter. On the postmortem examination, the overall prevalence of 20% (44/220) was infected with hydatid cyst. The prevalence of the condition was affected by sex, according to this study (*p* < 0.05). Of the 44 hydatidosis-positive animals, 27 (16.87%) were male, whereas 17 (28.8%) were female. Furthermore, male cattle were more affected (12.3%) than female cattle (7.7%), and this difference was statistically significant (*p* < 0.05). Apparently healthy cattle passed for slaughter; adult animals were shown to be more affected than old and young cattle in this study, with 10%, 7.3%, and 2.7%, respectively. According to this study, the prevalence of these diseases was affected by age with *p* < 0.05. 6 (10.9%) of the 44 hydatidosis-positive animals were young, 22 (19.8%) were adults, and 16 (29.63%) were old. The variation in prevalence by age was statistically significant (*p* < 0.05). Regarding body condition, the *p* value (*p* > 0.05) was statistically insignificant. Of the 44 hydatidosis-positive animals, 4 (13.79%) were lean, 17 (23.61%) were medium, and 23 (19.3%) were in good body condition. These findings show that there was no statistical significance of cattle origin with breed type (*p* > 0.05) (Table 1).

### 3.2. Cyst Characterization and Cyst Size Determination

Among the 44 visceral organs with positive harbored hydatid cysts, 27 (61.4%) were in the liver, 16 (36.3%) were in the lung, and 1 (2.3%) was found in the spleen (Table 2). Every cyst underwent a thorough growth examination and palpation for signs of calcification and degeneration. Measurements of cyst size, count, viability, and fertility were also carried out. A large cyst was predominantly found in the lung (25) (75.8%) than in the liver (*n* = 9) (81.8%), while a high number of small-sized and calcified cysts were found in the liver. The systemic measurement of cyst size indicated that high numbers of cysts of all sizes were found in the lung, liver, and spleen (Table 3). Cysts in the majority of animals with hydatidosis tended to be found in the liver and lungs rather than in other organs. 7 (22.6%) of the 34 cysts found on the liver were fertile, 8 (80.8%) were sterile, 4 (18.2%) were viable, and 19 (86.4%) were calcified. Of the 27 cysts found in the lungs, 23 (74.2%) were fertile, 1 (7.1%) was sterile, 18 (81.8%) were viable, and 3 (13.6%) were calcified. A solitary cyst in the spleen was found to be fertile. 31 (50%) of the 62 hydatid cysts collected and evaluated for fertility were fertile, 9 (14.5%) were sterile, and 22 (35.5%) were viable and calcified. Of the 31 fertile cysts, 23 (74.2%) were identified in the lung, while 7 (22.6%) and 1 (3.5%) were found in the liver and spleen, respectively (Table 4).

## 4. Discussion

The prevalence of bovine hydatidosis was 20% (44/220) in the current study. The findings of this study were comparable to the prevalence rates reported in previous studies, reported by 20% in Debre Markos [27] The findings of this study were comparable to the prevalence rates reported in previous studies, reported by 20% in Debre Markos [31], and 22.6% in Konso [32], and relatively similar findings of bovine hydatidosis have also been reported in neighboring countries, Morocco and Kenya, where the prevalence was reported to be 23% [33] and 19.4% [34]. However, Nebyou and Adugna [19] found that a prior report at Nekemte Municipal Abattoir (17.34%) was lower than the current study. Furthermore, a lower prevalence of bovine hydatidosis was observed throughout the country, with 11.26% in Mizan Teppi [35], 11.6% in Mekelle [36], and 16% in Wolaita Sodo [37]. In contrast, the current prevalence of hydatidosis was lower than that reported by authors in [38] in Hawassa (52.69%), [27], in Debre Markos (48.9%), and [39]in Bahir Dar (34.05%).

The variation in the incidence of hydatidosis could be due to demographic differences in definitive hosts (dogs), particularly stray dogs, and contact between them and intermediate hosts (cattle) [40]. Dogs were deployed as herd guards and are routinely fed uncooked offals that are judged unfit for human consumption, which is the primary source of disease transmission [41]. Another cause for differences in prevalence rates between countries and areas could be *E. granulosus* strain differences that exist in different geographical locations [42]. Other factors, such as differences in culture and social activities between locations, may also contribute to these differences [27].

The finding revealed that age and sex were potential risk variables. The infection rate increased as the cattle's age increased, and a statistical relationship was discovered between the age of the cattle and infection rate (*X*^2^ = 5.928, *p* = 0.015). This finding was consistent with that of [43], a study from the Ambo Municipal Abattoir. This could be because cattle were slaughtered at an older age, when they were more likely to be infected with *E. granulosus* [44]. The early culling of sick young calves through selling or killing before they reach old age could explain the decreased prevalence rate of hydatidosis in younger cattle [45]. Although hydatidosis affects both male and female, statistically significant differences were found in this investigation. Male animals had a higher rate of hydatid cyst infection than female animals (*X*^2^ = 4.086, *p* = 0.043), which could be attributed to the fact that the number of male animals sampled in the abattoir was approximately three times that of female animals.

The liver had the most calcified and tiny cysts, with 19 (86.4%) and 17 (94.4%), respectively. The liver (34) (54.8%) and the lungs (27) (43.5%) were both significant, which could be due to the liver's vast connective tissues and high number of reticuloendothelial cells [11]. Liver infection could be a reflection of the parasite's entrance pathway, and it appears to confirm the theory that oncosphere distribution in the hepatic portal leads to liver infection [46]. This discovery revealed that the most commonly affected organs were the lungs and liver. This was because the lung and liver have the first large capillaries met by migrating *Echinococcus* oncospheres (hexacanth embryos), which take the portal vein route and first pass through the hepatic and pulmonary filtration systems before moving on to any other peripheral organ [47]. However, migrating *Echinococcus* oncosphere*s* (hexacanth embryos) face bigger capillaries at the site of encounter in the lung and liver, which adopt moving blood-borne oncospheres and principally negotiate pulmonary and hepatic filtering systems sequentially before any other organs are involved. However, when the oncosphere escapes into the general systemic circulation, hydatid cysts develop in organs such as the spleen and other tissue organs [46].

The lung and soft spleen had the highest number of medium and large cysts compared to any other organ. This could be due to the lung tissues' soft nature, which makes it simpler for cyst pressure to develop [48, 49]. The current cyst fertility and viability findings of 31 (50%) fertile, 9 (14.5%) sterile, and 22 (35.5%) calcified were identical to previous findings of 50% [27]. The discrepancy in fertility rates among the different species could be attributable to differences in *E. granulosus* strains [42]. The lung has a greater fertility rate than the liver. This could be owing to the softer consistency of lung tissue, which permits cysts to form more easily [50]. The hydatid cyst's fertility rate may have a tendency to rise as the host's age rises [51].

The condemned organs and carcasses are buried in the Nekemte Municipal Abattoir [52], where some of them are later processed for use as animal feed. In dogs and other carnivores, this may lessen contamination and illness. Consequently, the lower incidence of hydatidosis in this study when compared to earlier research done across the nation may be the result of properly disposing of the condemned organ in a way that prevents dogs from accessing contaminated organs. The life cycle of *E. granulosus* in stray dogs and wild carnivores in the area is, however, maintained in part by backyard slaughter practices during local festivities, the custom of feeding uncooked infected offal to pet animals around homesteads, low public awareness of the diseases, and the practice of discarding dead domestic or wild animals unburied and left open for scavenging carnivores.

## 5. Conclusion and Recommendation

The study's overall somewhat high prevalence suggested that hydatidosis is a significant illness in the Nekemte area that is concerning for both public health and the economy. Hydatidosis is a significant parasitic disease in the area due to the high fertility and viability rates of hydatid cysts collected from the study area as well as the current socioeconomic conditions of the population. Both age and sex of the cattle were statistically related with the occurrence of bovine hydatidosis among the risk factors studied. The study found that bovine hydatidosis is a common and serious disease of cattle in the Nekemte region of Ethiopia, for which organ condemnation has great economic cost, and there are potential health hazards due to the lethal parasite. The overall prevalence rate of 20% shows that the disease is still a problem with regard to the population, especially older cattle and cattle with poor body conditions being the most affected. The finding of a great number of cysts in the liver and lung organs also indicates that improvements in the control measures are due. Judging from these results, it is suggested that the scope of anti-hydatidosis campaigns should be wide, including public education on the disease, strict laws for abattoir, and appropriate treatment of infected carcasses in order to control hydatidosis. Moreover, more works are needed to study *Echinococcus* species and develop strategies for their control, including treatment of stray dogs by deworming, and the community should be made aware of the diseases. In the town of Woreda, the government should be focusing on creating abattoirs with adequate facilities and regulating backyard slaughterhouses.

## Figures and Tables

**Table 1:** Hydatid cyst occurrence with various potential association risk factors.

Risk factor	No. of examined	No. of positive	Prevalence	*X* ^2^	*p* value
*Sex*					
Female	59	17 (28.8%)	7.7	4.086	0.043
Male	161	27 (16.87%)	12.3		

*Age*					
Young	55	6 (10.9%)	2.7	5.928	0.015
Adult	111	22 (19.8%)	10		
Old	54	16 (29.63%)	7.3		

*Body condition*					
Lean	29	4 (13.79%)	1.8		
Medium	72	17 (23.61%)	7.7	1.176	0.514
Good	119	23 (19.3%)	10.5		

*Origin*					
Bandra	32	7 (21.87%)	3.2		
W/Tuka	5	2 (40%)	1		
A/Gudatu	27	5 (18.51%)	2.3		
Guder	4	0	0		
Diga	3	1 (33.33%)	0.45	7.977	0.566
Ginchi	11	4 (36.36%)	1.8		
Arjo	12	1 (8.33%)	0.45		
Nekemte	2	1 (50%)	0.45		
Getema	2	0	0		
Chewaka	2	0	0		
D/Kane	3	0	0		
Uke	117	23 (19.65%)	10.5		

*Breed type*					
Local	208	41 (19.7%)	18.6	0.183	0.669
Exotic	12	3 (25%)	1.4		

**Table 2:** Distribution of hydatid cysts in different visceral organs at Nekemte Municipal Abattoir.

Organs	No. of positive	Percentage (%)
Liver	27	61.4
Lung	16	36.3
Spleen	1	2.3
Kidney and heart	0	0
Total	44	100

**Table 3:** Hydatid cyst size determination at Nekemte Municipal Abattoir.

Organs	No. of cysts	Cyst size		
		Small	Medium	Large
Liver	34	17 (94.4%)	9 (81.8%)	8 (24.2%)
Lung	27	1 (5.6%)	1 (9.1%)	25 (75.8%)
Spleen	1	0	1 (9.1%)	0
Total	62	18	11	33

**Table 4:** Hydatid cyst distribution and characterization at organ level.

Organs	Fertile	Sterile	Viable	Calcified	Total
Liver	7 (22.6%)	8 (88.9%)	4 (18.2%)	19 (86.4%)	34
Lung	23 (74.2%)	1 (11.1%)	18 (81.8%)	3 (13.6%)	27
Spleen	1 (3.2%)	0	0	0	1
Kidney and heart	0	0	0	0	0
Total	31	9	22	22	62

## Data Availability

All datasets that have led to the drawn deductions in the manuscript are herein presented in the paper, and upon reasonable request, the corresponding author can grant access to the datasets used and/or examined in this study.

## References

[1] Bizuwork A., Kebede N., Tibat T., Tilahun G., Kassa T. (2013). Occurrences and Financial Significance of Bovine Cystic Echinococcosis in Southern Wollo, Northeastern Ethiopia. *Journal of Veterinary Medicine and Animal Health*.

[2] Eckert J., Deplazes P., Craig P. S. (2001). Echinococcosis in Animals: Clinical Aspects, Diagnosis and Treatment. *WHO/OIE Manual on Echinococcosis in Humans and Animals: A Public Health Problem of Global Concern*.

[3] Budke C. M., Deplazes P., Torgerson P. R. (2006). Global Socioeconomic Impact of Cystic Echinococcosis. *Emerging Infectious Diseases*.

[4] Shuramo M. Y., Gutama K. P., Bulcha M. R., Pal M. Prevalence, Associated Risk Factors and Cyst Distribution of Hydatidosis in Cattle Slaughtered at Nekemte Municipal Abattoir. *Western Ethiopia*.

[5] Deplazes P., Jimenez-Palacios S., Gottstein B., Skaggs J., Eckert J. (1994). Detection of Echinococcus Coproantigens in Stray Dogs of Northern Spain. *Applied Parasitology*.

[6] Craig P. S., Gasser R. B., Parada L. (1995). Diagnosis of Canine Echinococcosis: Comparison of Coproantigen and Serum Antibody Tests With Arecoline Purgation in Uruguay. *Veterinary Parasitology*.

[7] Siles-Lucas M., Casulli A., Conraths F. J., Müller N. (2017). Laboratory Diagnosis of Echinococcus Spp. in Human Patients and Infected Animals. *Advances in Parasitology*.

[8] FAO (2011). *Manual on Meat Inspection for Developing Countries*.

[9] Eckert J., Deplazes P. (2004). Biological, Epidemiological, and Clinical Aspects of Echinococcosis, a Zoonosis of Increasing Concern. *Clinical Microbiology Reviews*.

[10] Parodi P. (2001). Public Health Education and Training in Control Programmes. *WHO/OIE Manual on Echinococcosis in Humans and Animals: A Public Health Problem of Global Concern*.

[11] Gemmell M. A., Roberts M. G., Beard T., Lawson J. R. (2001). Epidemiology: Quantitative Epidemiology and Transmission Dynamics With Special Reference to Echinococcus Granulosus. *WHO/OIE Manual on*.

[12] Gessese A. T. (2020). Review on Epidemiology and Public Health Significance of Hydatidosis. *Veterinary Medicine International*.

[13] Kassa S. A. (2012). Cystic Hydatidosis in Ethiopia: A Review. *Scientific Journal of Crop Science*.

[14] Asrate S. (2012). *Cystic Hydatidosis in Ethiopia: A Review*.

[15] Torgerson P. R., Dowling P. M., Abo-Shehada M. N. (2001). Estimating the Economic Effects of Cystic Echinococcosis. Part 3: Jordan, a Developing Country with Lower-Middle Income. *Annals of Tropical Medicine and Parasitology*.

[16] Jilo S. A., zakir Abadura S., Hussein J. A., Gelchu A. A. Prevalence and Economic Importance of Bovine Hydatidosis in Animal Slaughtered in Dalomana Municipal Abattoir Southeastern, Ethiopia.

[17] Nekemte Town Administration Office (2013). Annual Report.

[18] Thrusfield M. (2007). *Veterinary Epidemiology*.

[19] Nebyou M., Adugna D. (2014). Prevalence, Cyst Viability, Organ Distributions and Financial Losses Due to Hydatidosis in Cattle Slaughtered at Nekemte Municipal Abattoir, Western Ethiopia. *Journal of Veterinary Medicine and Animal Health*.

[20] Gracey J. F. (1986). *Meat Hygiene*.

[21] Nicholson M. J., Butterworth M. H. (1986). *A Guide to Condition Scoring of Zebu Cattle*.

[22] Lahunta A. D., Habel R. E. (1986). Applied Veterinary Anatomy.

[23] FAO https://www.fao.org/faostat/en/#data.

[24] Mellau B. L., Nonga H. E., Karimuribo E. D. (2011). Slaughter Stock Abattoir Survey of Carcasses and Organ/offal Condemnations in Arusha Region, Northern Tanzania. *Tropical Animal Health and Production*.

[25] Schantz P. M. (1991). Parasitic Zoonoses in Perspective. *International Journal for Parasitology*.

[26] Oostburg B. F., Vrede M. A., Bergen A. E. (2000). The Occurrence of Polycystic Echinococcosis in Suriname. *Annals of Tropical Medicine and Parasitology*.

[27] Kebede N., Abuhay A., Tilahun G., Wossene A. (2009). Financial Loss Estimation, Prevalence and Characterization of Hydatidosis of Cattle Slaughtered at Debre Markos Municipality Abattoir, Ethiopia. *Tropical Animal Health and Production*.

[28] Macpherson C. N., Zeyhle E., Romig T. (1984). An Echinococcus Pilot Control Programme for North-West Turkana, Kenya. *Annals of Tropical Medicine and Parasitology*.

[29] Kebede T., Zekarias T. (2020). Prevalence and Financial Loss Estimation of Hydatidosis of Cattle Slaughtered at Shashemene Municipal Abattoir, South Central Oromia, Ethiopia. *European Journal of Biological Sciences*.

[30] Dalimi A., Motamedi G. H., Hosseini M. (2002). Echinococcosis/Hydatidosis in Western Iran. *Veterinary Parasitology*.

[31] Moges E. (2003). *Study on Bovine Faciolosis and Hydatidosis at Jimma Abattoir*.

[32] Fikre L. (1994). Echinococcosis/Hydatidosis in Konso (Southern Ethiopia): An Assessment Trial of Its Prevalence, Economic and Public Health Importance.

[33] Azlaf R., Dakkak A. (2006). Epidemiological Study of the Cystic Echinococcosis in Morocco. *Veterinary Parasitology*.

[34] Njoroge E. M., Mbithi P. M., Gathuma J. M. (2002). A Study of Cystic Echinococcosis in Slaughter Animals in Three Selected Areas of Northern Turkana, Kenya. *Veterinary Parasitology*.

[35] Bekele J., Kebede W., Shimelis S., Shiferaw D. (2013). Prevalence and Financial Loss Estimation of Cystic Echinoccocosis in Cattle Slaughtered at Mizan Teferi and Teppi Municipal Abattoir South Western Ethiopia. *European Journal of Applied Sciences*.

[36] Yitbarek D. Y. D., Mulugeta Tefera M. T., Mihreteab Bekele M. B. Prevalence of Hydatidosis of Sheep Slaughtered at Abergelle Export Abattoir, Mekelle, Northern Ethiopia.

[37] Kebede N., Mekonnen H., Wossene A., Tilahun G. (2009). Hydatidosis of Slaughtered Cattle in Wolaita Sodo Abattoir, Southern Ethiopia. *Tropical Animal Health and Production*.

[38] Regassa F., Molla A., Bekele J. (2010). Study on the Prevalence of Cystic Hydatidosis and its Economic Significance in Cattle Slaughtered at Hawassa Municipal Abattoir, Ethiopia. *Tropical Animal Health and Production*.

[39] Kebede N., Mitiku A., Tilahun G. (2009). Hydatidosis of Slaughtered Animals in Bahir Dar Abattoir, Northwestern Ethiopia. *Tropical Animal Health and Production*.

[40] Bourée P. (2001). Hydatidosis: Dynamics of Transmission. *World Journal of Surgery*.

[41] Getaw A., Beyene D., Ayana D., Megersa B., Abunna F. J. (2010). Hydatidosis: Prevalence and its Economic Importance in Ruminants Slaughtered at Adama Municipal Abattoir, Central Oromia, Ethiopia. *Acta Tropica*.

[42] Arene F. O. (1985). Prevalence of Hydatid Cysts in Domestic Livestock in the Niger Delta. *Tropical Animal Health and Production*.

[43] Endrias Z., Yechale T., Assefa M. (2010). Bovine Hydatidosis in Ambo Municipality Abattoir, West Shoa, Ethiopia. *Ethiopian Veterinary Journal*.

[44] Assefa A., Tesfay H. (2013). Major Causes of Organ Condemnation and Economic Loss in Cattle Slaughtered at Adigrat Municipal Abattoir, Northern Ethiopia. *Hernia*.

[45] Zemen M., Bogale B., Derso S., Tassew A. (2015). Hydatidosis Prevalence, Cyst Viability and Organ Distribution and Economic Significance in Small Ruminants Slaughtered at Hashim Nur’s Export Abattoir, Debrezeit, Ethiopia. *Acta Parasitologica Globalis*.

[46] Urquhart G. M., Aremour J., Dunchan J. L., Dunn A. M., Jeninis F. W. (1996). *Veterinary Parasitology*.

[47] Mekuria S., Hirpassa L. (2015). Prevalence and Viability of Hydatidosis in Cattle Slaughtered at Sebeta Municipal Abattoir, Central Ethiopia. *Significance*.

[48] Hubert W. T. (1975). *Disease Transmitted from Animals to Man*.

[49] Smyth J. D. (1985). *Introduction to Animal Parasitology*.

[50] Assefa H., Mulate B., Nazir S., Alemayehu A. (2015). Cystic Echinococcosis Amongst Small Ruminants and Humans in Central Ethiopia. *Onderstepoort Journal of Veterinary Research*.

[51] Himonas C., Frydas S., Antoniadol-Sotiriadou K. (1987). The Fertility of Hydatid Cysts in Food Animals in Greece. *InHelminth Zoonoses*.

[52] Muhammed A., Tamiru Y., Kenei F., Zenu N. (2024). Prevalence of Bovine Hydatidosis in Cattle Slaughtered at Nekemte Municipal Abattoir, Western Ethiopia. *PREPRINT (Version 1)*.

